# *Persicariajucunda* var. *rotunda* (Polygonaceae, Persicarieae), a distinct distylous taxa raised to specific rank

**DOI:** 10.3897/phytokeys.126.35442

**Published:** 2019-07-16

**Authors:** Yue-Ning Guo, Shao-Feng Chen, Ming-Lin Chen, Bo Li

**Affiliations:** 1 School of Life Sciences, Nanchang University, Nanchang 330031, Jiangxi, China Nanchang University Nanchang China; 2 Provincial Key Laboratory of Biotic Environment and Ecological Safety in Anhui, Anhui Normal University, Wuhu 241000, Anhui, China Anhui Normal University Wuhu China; 3 Research Centre of Ecological Sciences, Jiangxi Agricultural University, Nanchang 330045, Jiangxi, China Jiangxi Agricultural University Nanchang China

**Keywords:** Distyly, micro-morphology, new combination, *Polygonum*, variety

## Abstract

Persicariajucunda(Meisn.)Migovar.rotunda (Z.Z.Zhou & Q.Y.Sun) Bo Li was originally published in the genus *Polygonum* L. and treated as a variety of *P.rotundum* Meisn. [≡*Persicariajucunda* (Meisn.) Migo]. After carefully comparing the macro- and micro-morphological characteristics of the achenes, leaf epidermis and tepals and the habitat between the variety and its typical variety, we confirmed that P.jucundavar.rotunda is clearly different from *P.jucunda* and should not be treated as a variety, but be raised to a specific rank as *P.rotunda* (Z.Z.Zhou & Q.Y.Sun) Bo Li. The species is distylous and could be easily distinguished from all other *Persicaria* taxa by a combination of morphological characters, such as completely decumbent leafless basal branches, almost sessile leaves, linear-lanceolate with rounded leaf bases, spicate, short and dense inflorescences, slender pedicels longer than bracts and dimorphic flowers and achenes. *P.rotunda* is endemic to several large wetlands of eastern China and usually occurs as one of the dominant species in some plant communities.

## Introduction

*Persicaria* (L.) Mill., after separation from the former polyphyletic genus *Polygonum* L., is currently placed within the tribe Persicarieae of Polygonaceae. The genus contains approximately 150 species of annual or perennial herbs and occurs mainly in temperate and subtropical regions of the Northern Hemisphere (Brandbyge 1993). *Persicaria* is characterised by having usually entire, ciliate or pectinate ocrea, many-flowered, spike-like or capitate inflorescences, 4–5-lobed tepals with trifid venation, 4–8 stamens, spheroidal pollen grains with reticulate exine and epidermis of pericarp with narrow rectangular cells and undulating anticlinal walls (Haraldson 1978, Ronse Decraene and Akeroyd 1988, Brandbyge 1993, Ronse Decraene et al. 2000). The genus has been subdivided into four sections on the basis of anatomical traits (Haraldson 1978), viz., sect. Persicaria, sect. Cephalophilon (Meisn.) H.Gross, sect. Echinocaulon (Meisn.) H.Gross and sect. Tovara (Adans) H.Gross, while Galasso et al. (2009) proposed to include another two sections, sect. AmphibiaTzvelev andsect.Truelloides Tzvelev, based on molecular phylogenetic studies. In *Persicaria*, a number of species have been observed or confirmed as distylous, such as *P.chinensis* (L.) H.Gross (≡*Polygonumchinense* L.) (Reddy et al. 1977), *P.japonica* (Meisn.) H.Gross (Hiratsuka and Nakao 1996), *P.jucunda* (Meisn.) Migo (≡*Polygonumrotundum* Meisn.) (Chen and Zhang 2010), *P.hastato-sagittatua* (Mak.) Nakai ex Mori (≡*Polygonumhastato-sagittatum* Mak.) (Chen 2012), *P.wugongshanensis* Bo Li (Li 2014) and P.odorata(Lour.)Sojáksubsp.conspicua (Nakai) Yonek. (Kong and Hong 2018). Distyly is a type of heterostyly which is characterised by the reciprocal placement of stigmas and anthers in two (distyly) or three (tristyly) floral morphs in a species (Lloyd and Webb 1992).

Persicariajucundavar.rotunda (Z.Z.Zhou & Q.Y.Sun) Bo Li (Li et al. 2013) was originally published in the genus *Polygonum* and treated as a variety of *P.rotundum* (≡*Persicariajucunda*) (Zhou et al. 2007). The variety resembles *P.rotundum* in having glabrous stems and prostrate at base, densely spicate inflorescences, pinkish tepals, slender pedicels longer than bracts and trigonous achenes, but differs from the typical variety in having truncate and linear-lanceolate leaf blades with barely noticeable petioles (Zhou et al. 2007). However, when conducting a micro-morphological study of Chinese *Persicaria* species, we found that there are a number of distinct differences between the two taxa, including the shape of epidermal cells of both leaf sides, the occurrence of stomata on adaxial leaf surface, the stomatal type of abaxial leaf surface and the sculpture of achene surface. After re-examining the macro-morphology and the habitat of the two taxa, we confirmed that P.jucundavar.rotunda is clearly different from *P.jucunda* and should not be treated as a variety, but be raised to a specific rank as *Persicariarotunda* (Z.Z.Zhou & Q.Y.Sun) Bo Li.

## Materials and Methods

The field investigations were carried out from 2014 to 2019. Fresh leaf materials and flowers of both *P.jucunda* and *P.rotunda* were collected and immediately fixed in FAA solution (formalin: acetic acid: alcohol = 18:1:1). The measurement of morphological characters was conducted based on both herbarium specimens (JXAU, acronym according to Thiers 2019) and living plants by using a micrometer and a stereomicroscope. To make a morphological comparison between *P.jucunda* and *P.rotunda*, the variability of four quantitative characters (leaf length, leaf width, number of leaf lateral vein pairs, inflorescence length) was evaluated using univariate statistics (box plots) by SPSS 11.5 statistical software package (SPSS Inc., Chicago, IL, USA). To confirm the distyly in *P.rotunda*, the height of stigmas and anthers were measured for a single flower removed from 30 individuals per style morph. Methodology follows Chen and Zhang (2010).

For light microscopy (LM) observation of leaf epidermis, samples were taken from the mature leaves fixed in FAA solution, dissected under a OPTPro stereoscope (Chongqing Optec Instrument Co. Ltd., China), stained in a solution of 1% safranin, and cleaned in distilled water three times before being mounted in glycerine jelly. Observations and micrographs were conducted randomly from 5 prepared slides per species under LM. Terminology follows Hou (2006).

For scanning electron microscopy (SEM) observations, samples of achenes were removed from mature fruits and dried in silica gel. After cleaned in 95% ethanol, mounted on to cupreous stubs and coated by JFC-1100E sputter coater (JEOL Led., Japan), samples were examined under JSM-6360LV SEM (JEOL Led., Japan) at a voltage of 25 KV. Terminology follows Ronse Decraene et al. (2000).

## Taxonomy

### Persicaria rotunda

Taxon classificationPlantaeCaryophyllalesPolygonaceae

(Z.Z.Zhou & Q.Y.Sun) Bo Li, comb. & stat. nov.

urn:lsid:ipni.org:names:77199242-1

Figures 1 , 2

≡PolygonumjucundumMeisn.var.rotundum Z.Z.Zhou & Q.Y.Sun, Acta Phytotax. Sin. 45(5): 714 (713–718; figs.). 2007. **Type**: CHINA. Anhui Province, Dongzhi County, Shengjin Lake, on riparian plains, Alt. 6–20 m, 4 October 2006, *Z.Z.Zhou 0602* (holotype: PE!, isotype: ANU).
≡Persicariajucunda(Meisn.)Migovar.rotunda (Z.Z.Zhou & Q.Y.Sun) Bo Li, Phytotaxa 91 (1): 24. 2013.

#### Diagnosis.

This species is easily distinguished from other *Persicaria* taxa by its completely decumbent leafless basal branches, almost sessile leaves, truncate, linear-lanceolate leaf blades, spicate, short and dense inflorescences, pedicels longer than bracts and dimorphic flowers and achenes. It occurs as one of the dominant species of some lakeshore plant communities in several large wetlands of northern Jiangxi and south-western Anhui provinces, eastern China.

#### Description.

*Annual herbs*. *Stems* slender, glabrous, basal branches 6–26, completely decumbent, 3–15 cm long, leafless, dark brown, producing numerous fibrous roots at each node; 3–12 additional flowering shoots branched from the upper nodes of each basal branch, 6–65 cm high, prostrate to erect, green to purplish-red, nodes inflated, purple. *Leaves* nearly sessile; leaf blades narrowly lanceolate to linear-lanceolate, 1.5–12.5 cm long, 0.3–1.3 cm wide, lateral veins 9–16 pairs, both surfaces glabrous, base round, apex acuminate, margin entire, shortly ciliate. *Ocrea* tubular, 4.5–11.5 mm long, membranous, sparsely appressed pubescent, apex truncate, fimbriate, cilia 2.2–5.3 mm long. *Inflorescence* terminal, erect, spicate, dense, 0.5–3.8 cm long; peduncle 3.5–6.5 cm long, glabrous; bracts purplish-red, funnel-shaped, sparsely pubescent, margin submembranous, shortly ciliate, each bract contains 4–7-flowers. *Pedicel* longer than bracts, 1.5–2.5 mm. *Flowers* dimorphic; perianth 5-parted, pinkish-white; long-styled flowers (called as L-morph) 3.9–4.4 mm long, stamens 8, 2.6–3.2 mm, styles 3, connate to below middle, 4.3–5.1 mm, exserted; short-styled flowers (called as S-morph) 3.7–4.2 mm long, stamens 8, 3.8–4.9 mm, exserted, styles 3, connate to below middle, 2.4–2.9 mm; stigmas capitate; nectaries 8, arranged at the base of ovary. *Achenes* included in persistent perianth, dimorphic; L-morph dark brown, ellipsoid, trigonous, base rounded to broadly cuneate, apex acuminate, surface opaque, densely reticular - pitted, 3.6–4.2 mm long, 2.1–2.3 mm wide; S-morph black, ovoid, trigonous, base broadly cuneate, apex acute, surface opaque, densely pitted, 2.9–3.3 mm long, 1.8–2.1 mm wide.

#### Phenology.

Flowering was observed from July to December and fruiting from late July to January.

#### Distribution and habitat.

**Figure 1.**
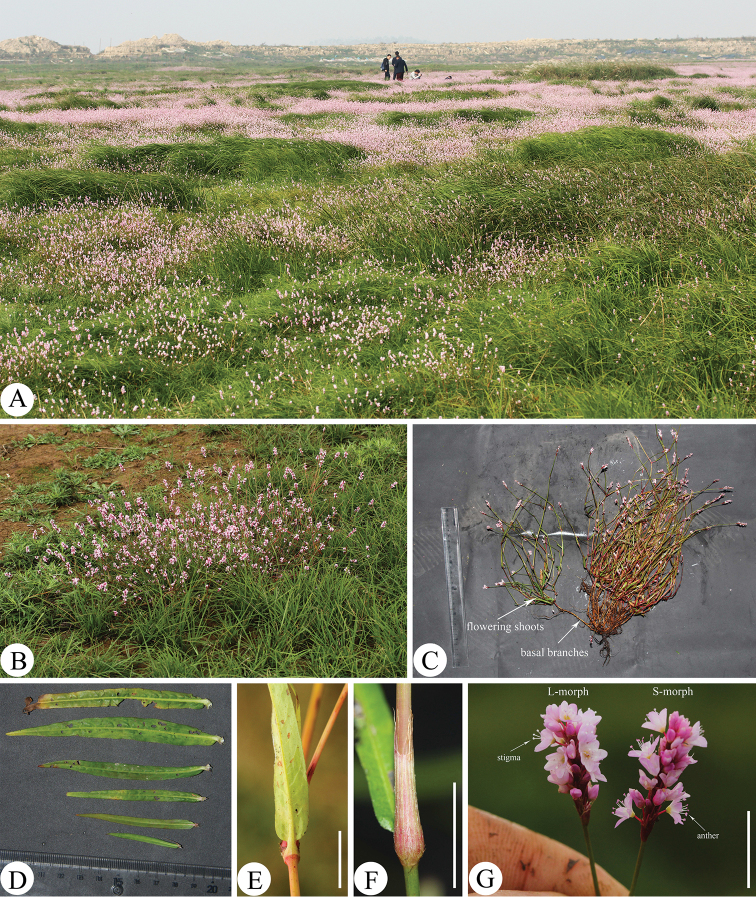
*Persicariarotunda* (Z.Z.Zhou & Q.Y.Sun) Bo Li **A** plant community with *P.rotunda***B** close-up view of an individual in situ **C** an individual showing branches **D** leaves **E** leaf base **F** ocrea **G** inflorescences. Scale bars: 1 cm (**E, F, G**).

*Persicairarotunda* is currently known only from several wetlands in north of Jiangxi Province and southwest of Anhui Province, eastern China and mainly grows in marshy and grassy areas around Daguan Lake, Lihu Lake, Longgan Lake, Poyang Lake Qingcao Lake, Shengjin Lake, Shimeng Lake and Wuchang Lake, which are several small to large lakes located near the Yangtze River. It usually occurs as one of the dominant species of some lakeshore plant communities (Fig. 1A).

**Figure 2.**
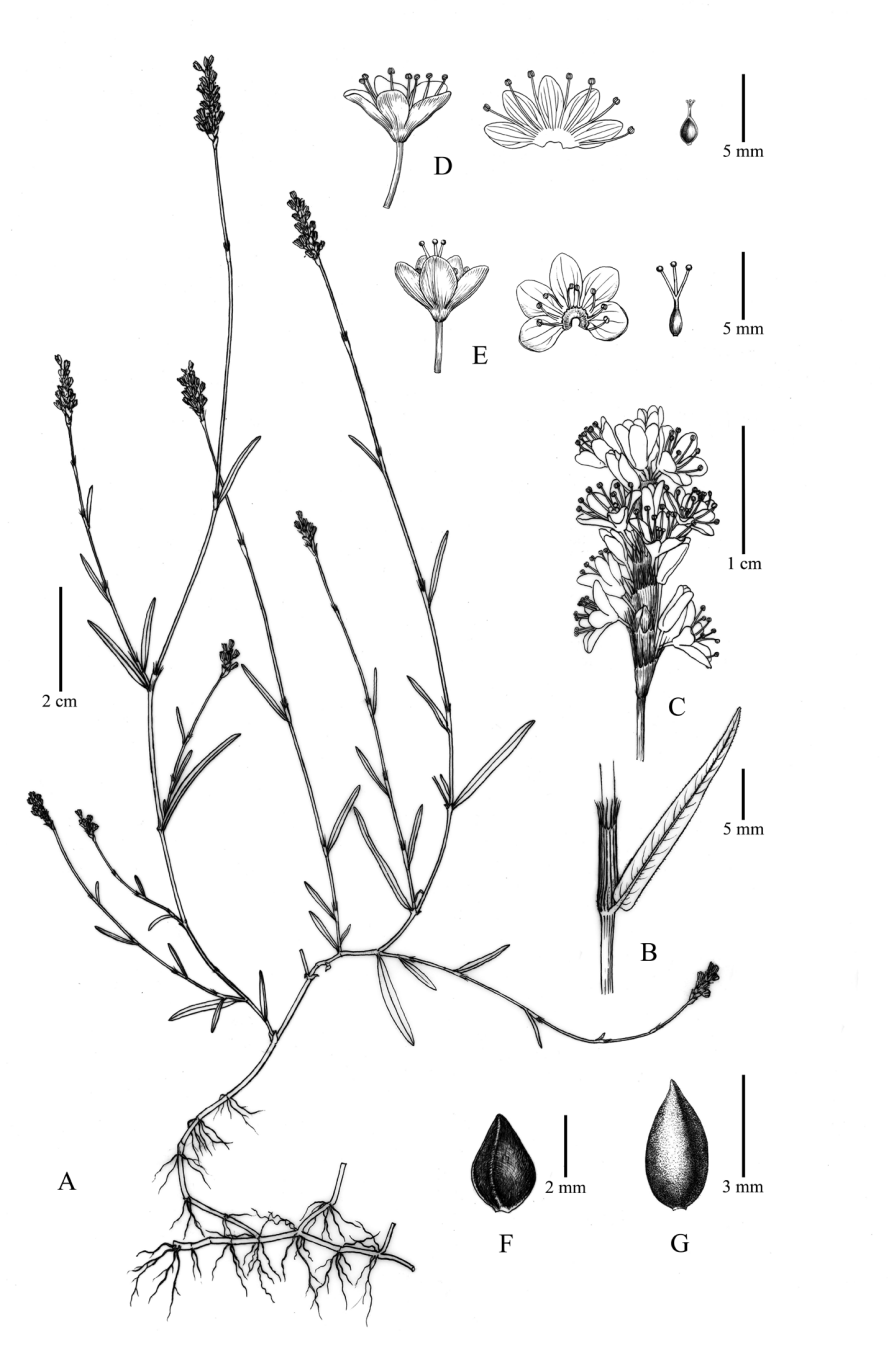
Line drawing of *Persicariarotunda* (Z.Z.Zhou & Q.Y.Sun) Bo Li **A** branches and inflorescences **B** ocrea and leaf **C** inflorescence **D** S-morph flower and its pistil **E** L-morph flower and its pistil **F** S-morph achene **G** L-morph achene.

#### Specimen examined.

CHINA. Jiangxi Province, Xingzi County, Shenling Lake, on grassy lakeside, Alt. 10 m, 29.270044N, 116.040173E, 16 July 2008, *B.Li JX046* (IBSC); Jiangxi Province, Yongxiu County, Wucheng Town, Poyang Lake, in wet meadow, Alt. 14 m, 29.114364N, 116.032021E, 11 December 2017, *B.Li LB0778* (JXAU); Jiangxi Province, Yongxiu County, Wucheng Town, Poyang Lake, in wetland marsh, Alt. 6 m, 29.133935N, 116.053571E, 15 October 2018, *B.Li LB0901* (JXAU).

#### Notes.

**Figure 3.**
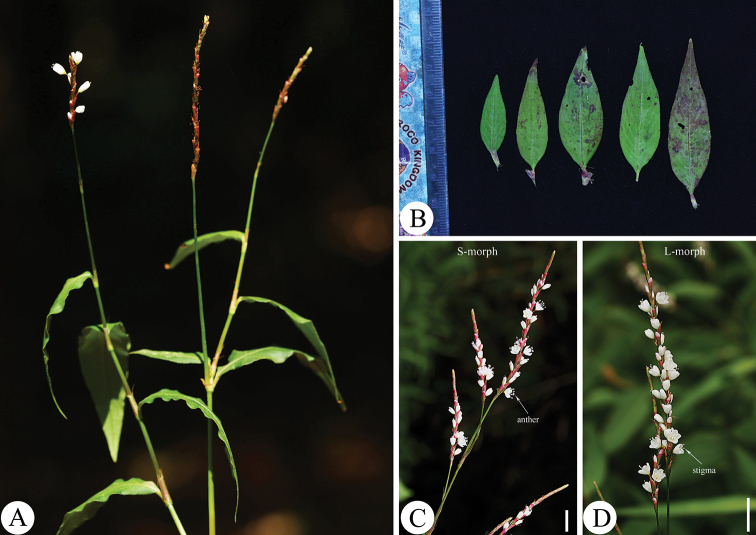
*Persicariajucunda* (Meisn.) Migo **A** habit **B** leaves **C** S-morph inflorescences **D** L-morph inflorescences. Scale bars: 1 cm (**C, D**).

**Figure 4.**
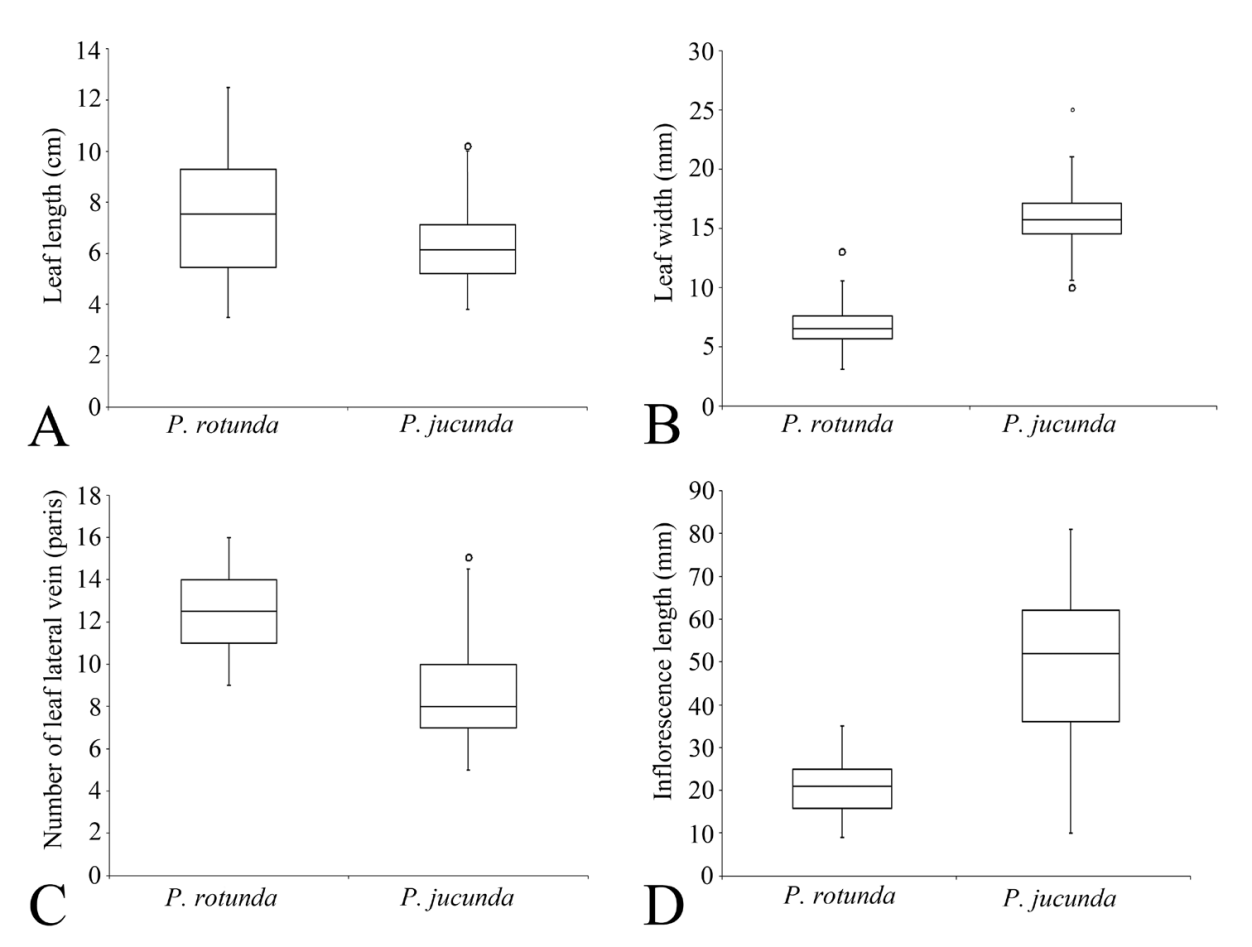
Box plots of four quantitative characters, leaf length (**A**) leaf width (**B**) number of leaf lateral vein pairs (**C**) and inflorescence length (**D**) of *Persicariarotunda* and *P.jucunda*. The boxes (rectangle region) represent the interquartile range and the whiskers (vertical line) represent the range excluding the outliers (circles). The three upper, middle and lower lines on the boxes represent the 75%, 50% and 25% of the variables, respectively. The upper and lower ends of the whiskers represent the maximum and minimum values of the variable, respectively. The circles represent the single value, where the variable value exceeds 1.5 times the difference between the 75% and 25%.

As noted by Zhou et al. (2007), *P.rotunda* is most similar to *P.jucunda* (Fig. 3) in gross morphology, particularly in having uninterrupted spicate inflorescences with dense flowers and slender pedicels longer than bracts. However, the authors did not notice that both of the species are distylous, which is another important similarity between the two taxa. The distyly of *P.jucunda* was firstly observed and confirmed by Chen and Zhang (2010). In the present study, we confirmed that *P.rotunda* is also a typical distylous species. The heights of the stigmas (4.63 ± 0.191 mm vs. 2.61 ± 0.056 mm, L-morph vs. S-morph) and anthers (2.74 ± 0.092 mm vs. 4.68 ± 0.178 mm, L-morph vs. S-morph) are reciprocal in the two morphs. However, *P.rotunda* is clearly different from *P.jucunda*, not only in some morphological traits (Fig. 4), but also in several micro-morphological characters (Table 1).

**Table 1.** Differences between *Persicariarotunda* and *P.jucunda*.

		*** Persicaria rotunda ***	*** P. jucunda ***
**Habitat**		wetlands	forest margins, grassy slopes or moist valleys
**Branches**		the primary branches completely decumbent, leafless and the secondary branches prostrate to erect	ascending to erect, without leafless branches
**Leaves**	Petiole	nearly absent	3–6 mm long
	Shape	narrowly lanceolate to linear-lanceolate	lanceolate or elliptic-lanceolate
	Lateral veins (pairs)	9–16	6–10
	Adaxial epidermis	polygonal epidermal cells with straight anticlinal walls no stomata no glands	irregular epidermal cells with straight to curved anticlinal walls stomata mostly anisocytic or occasionally paracytic sparse two-celled peltate glands
	Abaxial epidermis	irregular epidermal cells with curved to sinuolate anticlinal walls stomata anisocytic plenty of four-celled peltate and spheroidal glands	irregular epidermal cells with sinuolate to sinuate anticlinal walls stomata paracytic no glands
**Length of Inflorescences (cm)**		0.5–3.8	1.0–8.2
**Tepals**	Length (mm)	L-morph 3.9–4.4, S-morph 3.7–4.2	L-morph 2.8–3.3, S-morph: 2.7–3.2
	Epidermis	anticlinal walls of epidermal cells curved to sinuolate 10–14 sinuate striates on cuticular layer	anticlinal walls of epidermal cells sinuolate to sinuate 12–18 straight to sinuolate striates on cuticular layer
**Achenes**	Size (length × width, mm)	L-morph 3.6–4.2 × 2.1–2.3, S-morph 2.9–3.3 × 1.8–2.1	L-morph 2.1–2.6 × 1.6–1.8, S-morph 2.2–2.7 × 1.7–1.9
	Surface	opaque, densely pitted	shiny, smooth
	Epidermal ornamentations	reticulate	Indistinctly reticulate

Besides the differences summarised by Zhou et al. (2007), such as leaf shape, leaf width, petiole length and stem diameter, we observed several additional morphological traits that are clearly distinct between *P.rotunda* and *P.jucunda*. The stems of *P.rotunda* have 6–26 basal branches which are leafless and completely decumbent with numerous fibrous roots at each node. On the upper nodes of each basal branch, there are 3–12 flowering shoots which are prostrate to erect and normally bearing leaves and inflorescences (Fig. 1C). However, the stems of *P.jucunda* are mostly erect or only prostrate at the base and the number of its branches are much fewer than those of *P.rotunda*. *Persicariarotunda* also has more pairs of leaf lateral veins and much shorter inflorescences than *P.jucunda* (Fig. 4).

**Figure 5.**
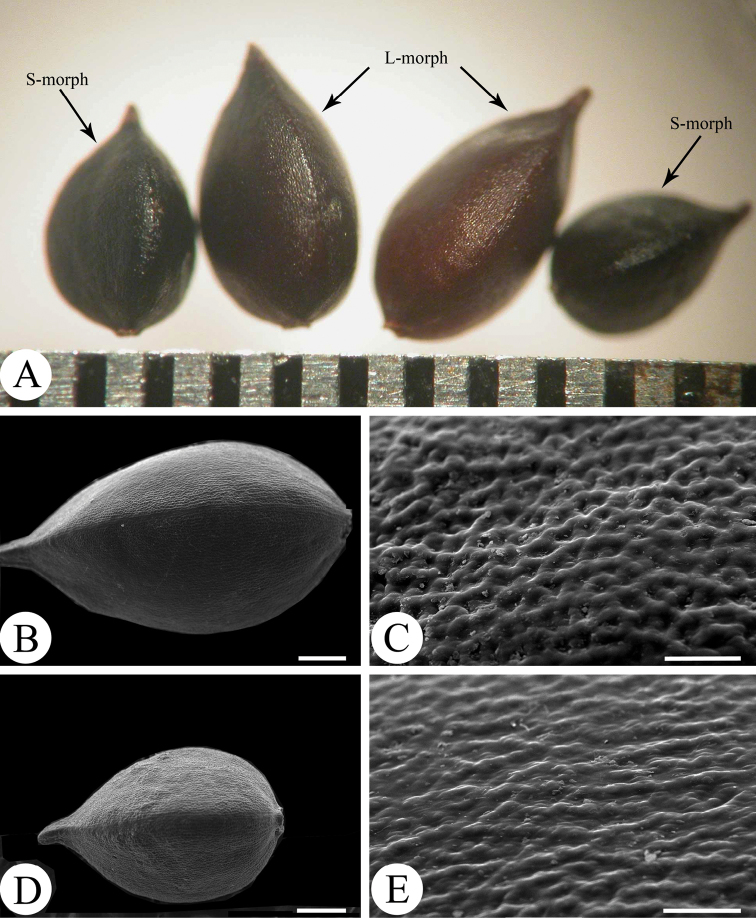
Achene morphology of *Persicariarotunda***A** dimorphic achenes under a stereoscope **B–C** SEM micrograph of L-morph achene **B** whole view **C** partial view showing its epidermis sculpture **D–E** SEM micrograph of S-morph achene **D** whole view **E** partial view showing its epidermis sculpture. Scale bars: 500 μm (**B, D**); 50 μm (**C, E**).

Though both of *P.rotunda* and *P.jucunda* have dimorphic flowers, the achenes of *P.rotunda* are also dimorphic, with the L-morph ellipsoid in shape and dark brown in colour, whereas the S-morph achene is ovoid in shape and black in colour (Fig. 5A). Additionally, the L-morph achenes of *P.rotunda* have larger size and more raised reticulate epidermal ornamentations than those of the S-morph (Figs 5B–E). In contrast, the achenes of *P.jucunda* are homomorphic with the same smooth surfaces in both morphs (Chen and Zhang 2010). Amongst the distylous taxa reported in *Persicaria*, *P.rotunda* is, so far, the only species that shows dimorphic features on achenes.

**Figure 6.**
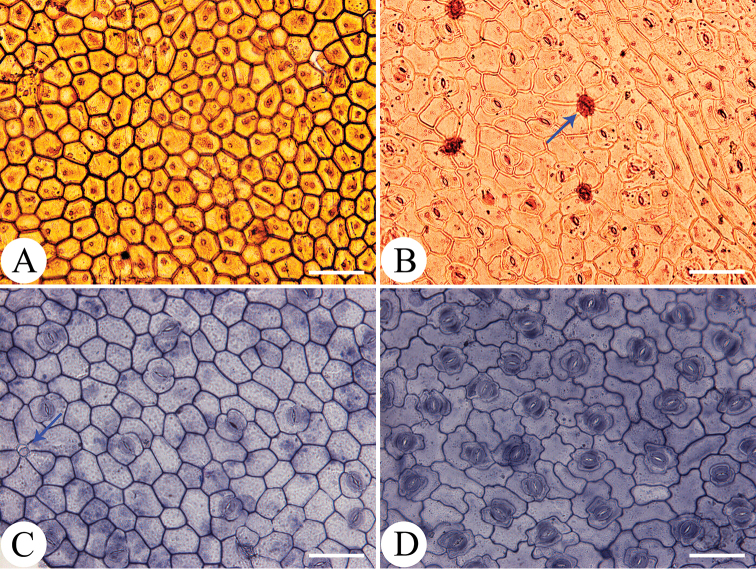
LM micrographs of leaf epidermis of *Persicariarotunda* (**A, B**) and *P.jucunda* (**C, D**). **A, C** upper epidermis **B, D** lower epidermis. Arrow in B shows the four-celled peltate and spheroidal glands of *P.rotunda* and in **C** indicates the two-celled peltate glands of *P.jucunda*. Scale bars: 500 μm.

Leaf epidermis characters have been tested to be of important taxonomic significance in *Persicaria* (Hou 2006, Zhu et al. 2007, Yasmin et al. 2010). When observing the leaf epidermis of *P.rotunda* and *P.jucunda*, we found that there are significant differences in the leaf epidermal micro-morphology of the two taxa (Fig. 6). In *P.rotunda*, the adaxial leaf epidermal cells are polygonal in shape with the straight anticlinal walls and no stomatal apparatus or gland occurs on the surface (Fig. 6A). However, the adaxial leaf epidermis of *P.jucunda* is covered by irregular epidermal cells with the anticlinal walls straight to curved and has mostly anisocytic or occasionally paracytic stomata and sparsely two-celled peltate glands (Fig. 6C). On the abaxial leaf epidermis, plenty of four-celled peltate and spheroidal glands, anisocytic stomata and irregular epidermal cells with the anticlinal walls curved to sinuolate were observed for *P.rotunda* (Fig. 6B), while in *P.jucunda*, no glands have been found, the stomata are paracytic and the anticlinal walls of epidermal cells are sinuolate to sinuate (Fig. 6D).

In the protologue, Zhou et al. (2007) also investigated the tepal micro-characteristics of *P.rotunda* and *P.jucunda* and listed their differences: the anticlinal walls of epidermal cells are curved to sinuolate in *P.rotunda*, while sinuolate to sinuate in *P.jucunda*; the cuticular layer has longitudinally 10–14 of sinuate striates in *P.rotunda*, while 12–18 straight to sinuolate striates in *P.jucunda*. Taking all the above morphological and micro-morphological evidence together, we think that *P.rotunda* represents a distinct species in *Persicaria* and it should not be placed under *P.jucunda* as a variety, but be treated as a separate species.

## Supplementary Material

XML Treatment for Persicaria rotunda
